# A Study of the Variation in the Salivary Peptide Profiles of Young Healthy Adults Acquired Using MALDI-TOF MS

**DOI:** 10.1371/journal.pone.0156707

**Published:** 2016-06-03

**Authors:** Andrei Prodan, Henk Brand, Sultan Imangaliyev, Evgeni Tsivtsivadze, Fridus van der Weijden, Ad de Jong, Armand Paauw, Wim Crielaard, Bart Keijser, Enno Veerman

**Affiliations:** 1 Top Institute Food and Nutrition, Wageningen, The Netherlands; 2 Department of Oral Biochemistry, Academic Center for Dentistry Amsterdam (ACTA) University of Amsterdam and Free University Amsterdam, Amsterdam, The Netherlands; 3 MSB Group, The Netherlands Organization for Applied Scientific Research (TNO), Zeist, The Netherlands; 4 Department of Periodontology, Academic Centre for Dentistry Amsterdam (ACTA), University of Amsterdam and Free University Amsterdam, Amsterdam, The Netherlands; 5 Department CBRN Protection, The Netherlands Organization for Applied Scientific Research (TNO), Rijswijk, The Netherlands; 6 Department of Preventive Dentistry, Academic Centre for Dentistry Amsterdam (ACTA), University of Amsterdam and Free University Amsterdam, Amsterdam, The Netherlands; Moffitt Cancer Center, UNITED STATES

## Abstract

A cross-sectional observational study was conducted to evaluate the inter-individual variation in the MALDI-TOF MS peptide profiles of unstimulated whole saliva in a population of 268 systemically healthy adults aged 18–30 yr (150 males and 118 females) with no apparent caries lesions or periodontal disease. Using Spectral Clustering, four subgroups of individuals were identified within the study population. These subgroups were delimited by the pattern of variation in 9 peaks detected in the 2–15 kDa m/z range. An Unsupervised Feature Selection algorithm showed that P-C peptide, a 44 residue-long salivary acidic proline-rich protein, and three of its fragments (Fr. 1–25, Fr. 15–35 and Fr. 15–44) play a central role in delimiting the subgroups. Significant differences were found in the salivary biochemistry of the subgroups with regard to lysozyme and chitinase, two enzymes that are part of the salivary innate defense system (p < 0.001). These results suggest that MALDI-TOF MS salivary peptide profiles may relate information on the underlying state of the oral ecosystem and may provide a useful reference for salivary disease biomarker discovery studies.

## Introduction

Saliva is crucial for the maintenance of oral health [1]. Salivary components provide lubrication, stabilize oral pH, aid remineralization of dental enamel and modulate growth and adherence of oral bacteria to tooth surfaces [2, 3].

The diagnostic potential of saliva has been increasingly explored during the last decades. A number of features make saliva an attractive medium for biomarker discovery [4–6]. First of all, saliva sampling is a non-invasive, safe, and cost-effective option compared to the collection of other body fluids. Secondly, salivary biomarkers not only give insight into the health status of the oral cavity, but can also convey information regarding systemic health. This is due to the presence of numerous serum-derived compounds which enter the oral fluid either directly (from the salivary glands) or indirectly (either via the gingival crevicular fluid or through inflamed gingiva or damaged parts of the oral mucosa) [5, 7, 8].

Saliva as a potential source of biomarkers poses its own unique set of challenges. Saliva is a mixture of secretions from three pairs of major glands (parotid, submandibular and sublingual) as well as numerous minor glands, each having a characteristic protein composition [9, 10]. As a result, the composition of whole saliva depends heavily on the manner in which saliva was collected and is also affected by factors such as age, sex, medication, circadian rhythm, physical activity, and oral hygiene procedures prior to collection [9, 11–13]. Careful standardization of saliva collection is therefore crucial for obtaining reproducible results [7]. Other complicating factors are related to the nature of salivary proteins and peptides. Firstly, virtually all the major salivary protein families display a large degree of phenotypic variation due to genetic polymorphisms, alternative RNA splicing and various post-translational modifications [9, 14]. Moreover, secreted saliva is exposed to proteolytic activity from both endogenous proteases (originating from the salivary glands or mucosal cells) and exogenous proteases (produced by the oral microflora) [9]. As the vast majority of salivary biomarkers are proteins / peptides, these additional levels of complexity hamper interpretation of the data.

Comparison of a healthy control group to a diseased group is common in studies that attempt to discover salivary biomarkers [15, 16]. This raises the question: what is the inherent biological variation of potential peptide biomarkers in the saliva of a healthy population? Is the variation among individuals related to specific sets of salivary peptides that can cluster individuals into discrete subgroups? Knowledge of the variation in peptide profiles in healthy saliva is therefore particularly relevant for salivary biomarker discovery.

The aim of this study was to examine inter-individual variation in MALDI-TOF MS salivary peptide profiles within a population of systemically healthy young adults and to identify potential subgroups.

## Materials and Methods

### Clinical study structure, study population and exclusion criteria

The study was carried out within the framework of the Top Institute Food and Nutrition project "Estimating the boundaries of a healthy oral ecosystem in young individuals" [17].

Whole unstimulated saliva was collected in a cross-sectional single-center observational clinical study at the Academic Center for Dentistry Amsterdam (ACTA). The protein biochemistry of the same saliva sample set has already been analyzed and described previously [17]. The study population comprised a convenience sample of systemically healthy young adults aged 18–30 yr old without periodontitis or apparent caries lesions. Participants were students of universities and colleges in and around Amsterdam, The Netherlands. They were invited for screening when they had visited a dentist the previous year and had been considered to be without oral or dental problems. The volunteers were screened for suitability according to the criteria of the Dutch Periodontal Screening Index (DPSI) [18]. They were included if they had a DPSI ≤ 3-. The following exclusion criteria were used: overt dental caries, inter-proximal restorations between the first and second or second and third upper molars, apparent oral lesions, infections, a habit of smoking, recent use of antibiotics, use of anti-inflammatory drugs or other prescribed medication which could interfere with the outcome of this study (except for oral contraceptives). Participants were instructed to abstain from eating, drinking, chewing gum or performing strenuous physical exercise prior to the appointment, and not to brush their teeth in the 24 hours prior to the appointment.

The study was conducted in accordance with the Declaration of Helsinki (2008) of the World Medical Association and approximated Good Clinical Practice guidelines. The study protocol was reviewed and approved by the Medical Ethics Committee of the Academic Medical Centre of Amsterdam (2012_210#B2012406) and recorded at the Dutch Trial Register (NTR3649). All participants signed an informed consent form.

### Unstimulated saliva sampling and salivary biochemical analysis

All saliva samples were collected between 9 and 10 a.m. Participants were instructed to accumulate saliva in the floor of the mouth without stimulation by orofacial movements and to spit at 30 s intervals into pre-weighed 30-ml polypropylene tubes (Sterilin, Newport, U.K.) which were kept on ice. The collection period was 5 min. The tubes containing unstimulated saliva samples were weighed and salivary flow rate was calculated assuming a saliva density of 1.0 g ml^-1^. Saliva samples were homogenized by vortexing for 20 s. Salivary pH and buffered pH were measured immediately after saliva collection as previously described [17]. Samples were clarified by centrifugation for 10 min at 4°C and 10,000 *g* to remove epithelial cell debris, bacteria and food residues. The resulting clarified saliva sample was diluted 1:1 with a 500 mM NaCl solution to a final concentration of 250 mM NaCl, aliquoted and stored at -80°C. The dilution prevented protein aggregation and precipitation during saliva freezing and storage and lowered viscosity allowing for more precise sample manipulation and improved reproducibility [17]. A sufficient number of aliquots of each sample were produced to avoid exposing samples to multiple freezing and thawing cycles. Salivary total protein content, mucins MUC5B and MUC7, lactoferrin, secretory-IgA, albumin, amylase, chitinase, proteases and lysozyme were measured as described previously [17].

### MALDI-TOF MS spectra acquisition

Prior to MALDI-TOF MS analysis the saliva samples were desalted using C18 ZipTips (Merck Millipore, Darmstadt, Germany) as follows: ZipTips were wetted twice with 10 μL acetonitrile (ACN), equilibrated 3 times with 0.1% trifluoroacetic acid (TFA), loaded with 10 μL of saliva sample diluted 1:50 with double-distilled water, and washed 3 times with 0.1% TFA. The purified peptides were then eluted and spotted directly onto a MALDI target plate with 1 μL of matrix solution (10 mg α-cyano-4-hydroxy cinnamic acid in 1 ml of ACN/water 1:1 (v/v) with 2.5% TFA). The spots were allowed to air-dry and spectra were acquired using an Autoflex III MALDI-TOF mass spectrometer (Bruker, Bremen, Germany). Spectra were recorded in linear mode at a mass range of 2–15 kDa with a 200 Hz laser at 355 nm. Each spectrum was an average of 2000 laser shots. The mass spectrometer was calibrated using a Bacterial Test Standard (Bruker, Bremen, Germany).

Samples were analyzed in duplicate, in 12 sessions spread over a period of one month. For duplicate analysis, separate aliquots of each saliva sample were analyzed independently (from storage to ZipTip-ing, spotting and spectral acquisition). Visual quality control was performed for all raw duplicate spectra. Samples were re-analyzed in new duplicates if either one of the duplicate spectra from a sample showed excessive noise or large baseline drift, or if the duplicate spectra exhibited dissimilarities. Spectra from 49 of the 261 samples analyzed (19%) failed initial quality control and were re-analyzed.

Spectra of a reference saliva sample were acquired in quadruplicate at each session in order to aid the subsequent sample spectral alignment and to assess the reproducibility of the assay. Peak intensity data from a set of 11 reference peaks in the mass-to-charge (m/z) range 2.0 to 7.6 kDa were used to calculate the within-session, between-session and overall coefficients of variation (CV). The mean within-session CV was 6% (range 4–12) while mean between-session CV was 10% (range 4–21, with the highest CV for the highest m/z peak considered, at 7.6 kDa). Overall CV was 12% (range 6–21).

### MALDI-TOF MS spectra processing

The raw MALDI-TOF MS spectra were processed in Matlab R2012b using the Mathworks bioinformatics tool box (MathWorks, Natick, MA, U.S.A). The workflow consisted of spectra resampling followed by baseline subtraction, smoothing and normalization of the total area under the curve (i.e. normalizing based on the total amount of sample protein ionized per spectrum). Normalization for total area under the curve insures that differences in saliva sample protein concentration, or in the protein concentration of the spotted sample, are compensated for. Reference spectra were used to align batches of spectra analyzed on different days and to compensate for inter-session instrument drift. Duplicate spectra were then averaged into one sample spectrum. Subsequently, peak detection was performed, followed by peak binning (peak coalescing) using a hierarchical clustering algorithm to calculate a common m/z reference peak vector. The final result was a 3-dimensional database of salivary peptide profiles consisting of sample ID’s, peak m/z values, and peak intensities. Peak identification was attempted using MALDI-MS/MS or by matching the peak m/z ratios to literature values from previous studies [19, 20]. The spectra processing workflow is exemplified in S1 Fig.

### MALDI-TOF MS/MS

Identification of MALDI-TOF peaks by MALDI-TOF MS/MS was attempted for all peaks <4,000 Da using a Laser Induced Fragmentation in Time (LIFT) protocol on a Autoflex III MALDI-TOF mass spectrometer (Bruker, Bremen, Germany). Spectra were averages of 8,000 laser shots. The mass window was ±13 Da around the precursor ion mass. The reflector detector voltage was 1.8 kV. For each individual precursor ion mass, the respective extraction delay times in both ion sources were calculated by the instrument control software.

### Spectral Clustering and Feature Selection

Spectral Clustering analysis was performed using the dataset that contained the processed data from all MALDI-TOF MS spectra. Spectral Clustering was performed in open-source Python 2.7 using the neighborhood co-regularized Spectral Clustering algorithm developed by Tsivtsivadze et al. [21] based on the Spectral Clustering method published by von Luxburg [22]. For this purpose, the data for each MALDI-TOF MS peak were scaled to equal ranges and a similarity matrix was calculated based on the Euclidean distances between each pair of participants (i.e. on the similarity of the overall peptide profiles of each pair of participants). A co-occurrence matrix was subsequently calculated based on the clustering results, quantifying the tendency of any pair of participants to fall within the same cluster over many k-means clusterings using varying parameters. The co-occurrence clustering plots were constructed using Matlab R2012b (MathWorks, Natick, MA, U.S.A). After visual examination of the Spectral Clustering plots the number of clusters was determined and participants were mathematically assigned to the clusters using a probabilistic decomposition algorithm [23].

Unsupervised Feature Selection was implemented in open-source Python 2.7 code using the Unsupervised Multi-View Feature Selection via Co-Regularization algorithm [24]. Data from all features in the input dataset (i.e. intensities corresponding to each m/z value) were scaled to equal range. The output of Unsupervised Feature Selection was a list of features ranked based on the values of the weighting coefficients’ vector norm of the regularized spectral regression problem. The highest ranking scores based on the vector norm values correspond to the features that were the most important in determining the data clustering [24].

### Data analysis and statistical methods

The data were analyzed statistically using SPSS 21.0 software (IBM, Armonk, NY, U.S.A.). Pearson’s product-moment was used to assess correlations and independent sample t-tests were used to compare means. The statistical significance level used was 0.05. The Benjamini-Hochberg False Discovery Rate (FDR) procedure was used to correct for multiple comparisons [25]. The FDR was set at 0.05. Cohen’s d was used to quantify effect size [26].

## Results

Prior to the study, 336 potential participants were screened at the dental clinic in a separate session. Of these, 46 (23 males and 23 females) were excluded based on pre-defined inclusion and exclusion criteria as reported in a previous publication (16). Of the remaining 290 subjects, 10 took part in a pilot study (not included in the final data) and 12 dropped out (11 due to schedule conflicts and one reported pregnancy). In total, 268 participants completed the study (150 males, 118 females), with a mean age of 22.6 yr (on the day of their appointment) and a range of 18–32 yr. Of these, 7 were unable to provide sufficient saliva during the sampling period. Data obtained from the remaining 261 participants (145 male and 116 female) are presented in this article.

A total of 129 peaks were detected in the MALDI-TOF MS spectra. Of these, 49 peaks were present in less than 2% of the samples (i.e. in less than 5 participants out of 261). These low frequency peaks were filtered out and all further analyses were performed on the remaining 80 peaks. The complete dataset is available in S1 Dataset. Table 1 lists the peaks for which either definitive or putative identities were assigned. One peak was identified by means of MALDI-TOF MS/MS: a peak at 2043 Da was identified as a P-C peptide fragment containing amino acids 15 through 35 (Fr. 15–35). The MS/MS spectrum is shown in S2 Fig. Another 19 peaks were identified by matching their m/z values to those reported in previous studies [19, 27].

**Table 1:** Putative identities for peaks in the MALDI-TOF MS spectra of saliva from healthy volunteers.

m/z [Da]	Present in [%] of individuals	Peak identity
**2043**	92	P-C peptide Fr. 15–35^a^
**2185**	95	P-C peptide (2+ charge peak)^b^
**2523**	94	P-C peptide Fr. 1–25^b^
**2921**	94	P-C peptide Fr. 15–44^b^
**3039**	42	Histatin 3 Fr. 1–24 (Histatin 5)^b^
**3200**	18	Histatin 3 Fr. 1–25^c^
**3376**	5	α-defensin 2^b^
**3447**	8	α-defensin 1^b^
**3497**	88	α-defensin 3^b^
**4063**	8	Histatin 3^b^
**4250**	23	P-C peptide Des Q44^b^
**4371**	69	P-C peptide (1+ charge peak)^b^
**4557**	8	P-B Des 1–12^c^
**4921**	26	Histatin 1^b^
**5390**	8	Statherin diphosporylated^b^
**5954**	79	Cystatin B Fr. 1–53^b^
**7528**	12	II-2 basic proline rich protein^b^
**7618**	66	II-2 basic proline rich protein, phosporylated^b^
**10444**	17	S100A12 (calgranulin C)^b^
**11172**	26	aPRP-4, diphosph./PRP-3 diphosphorylated^b^

^a^Identification by MALDI-TOF MS/MS (S2 Fig).

^b^Castagnola *et al*. 2012 [27].

^c^Cabras *et al*. 2010 [19].

**Fig 1:**
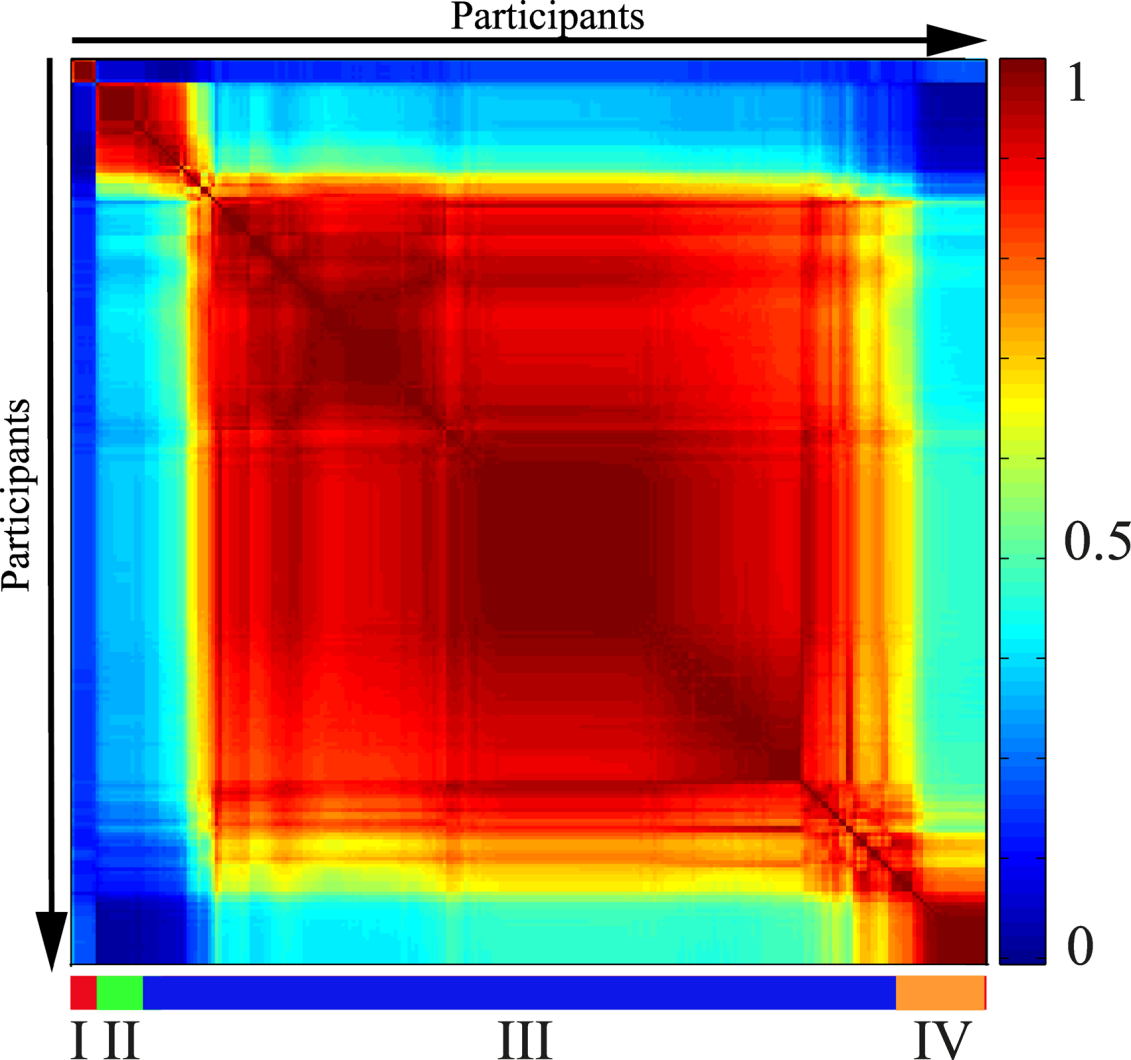
Spectral Clustering co-occurrence plot. Participants are ordered along both X- and Y-axis according to the co-occurrence score (i.e. the more similar the peptide profiles of any two participants, the higher their tendency to cluster together and the closer they are placed on the axis). Co-occurrence score values range from 0 (for participants who never cluster together) to 1.0 (for participants who always cluster together). The horizontal bar delimits the four clusters.

**Fig 2:**
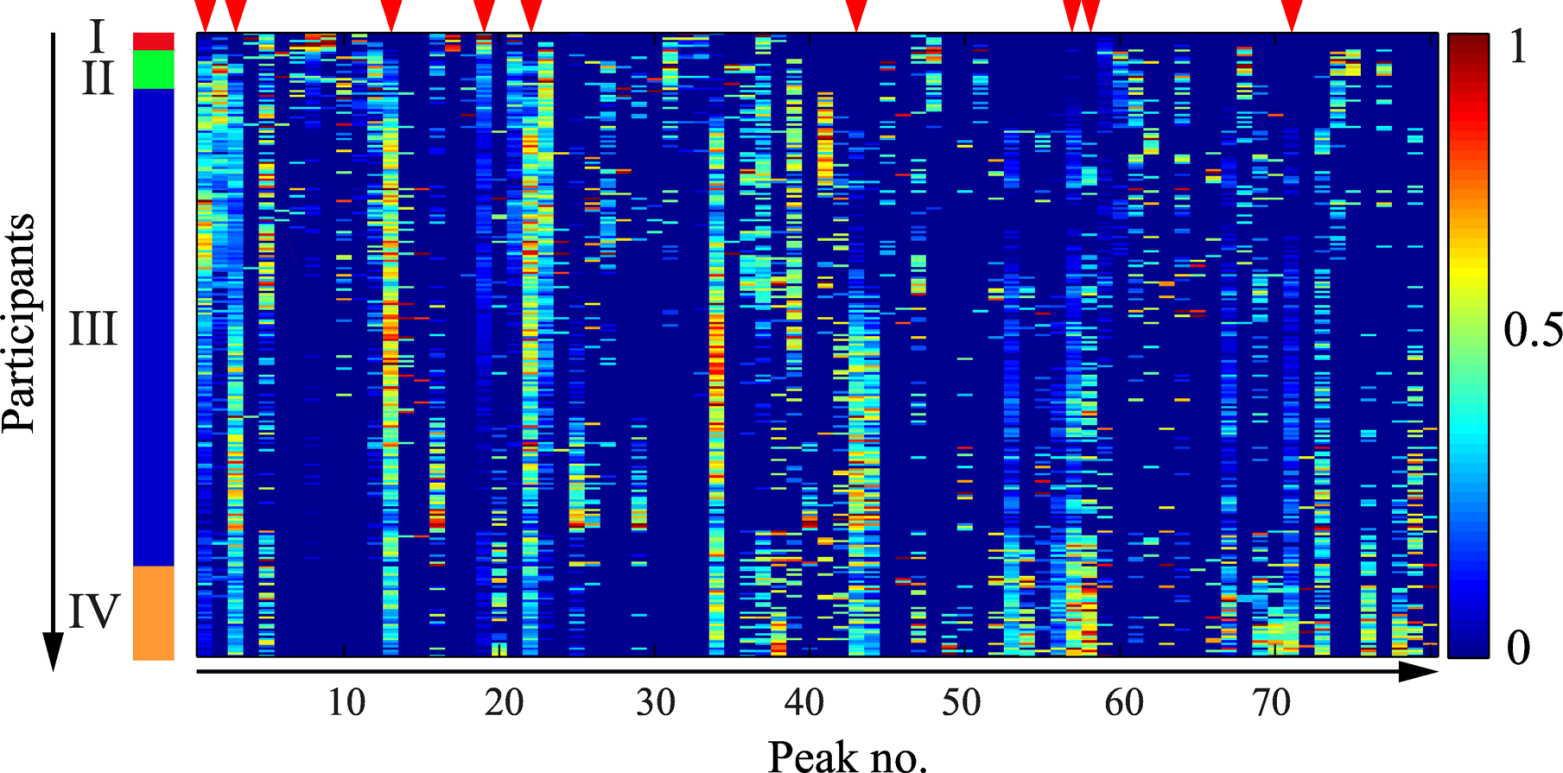
Overview of the peptide profile variation within the study population. The X-axis contains the 80 peaks ordered by their m/z ratio, (lowest to highest, from left to right). The Y-axis contains the 261 participants, arranged according to the co-occurrence matrix (same as in Fig 1). Data for each individual peak were scaled to equal range (from 0 to 1) and color coded (legend on the right side of the figure). The vertical bar on the left delimits the four clusters. Red arrows on the upper margin highlight the 9 peaks shown by Unsupervised Feature Selection to be determining the clustering structure (Fig 1).

**Fig 3:**
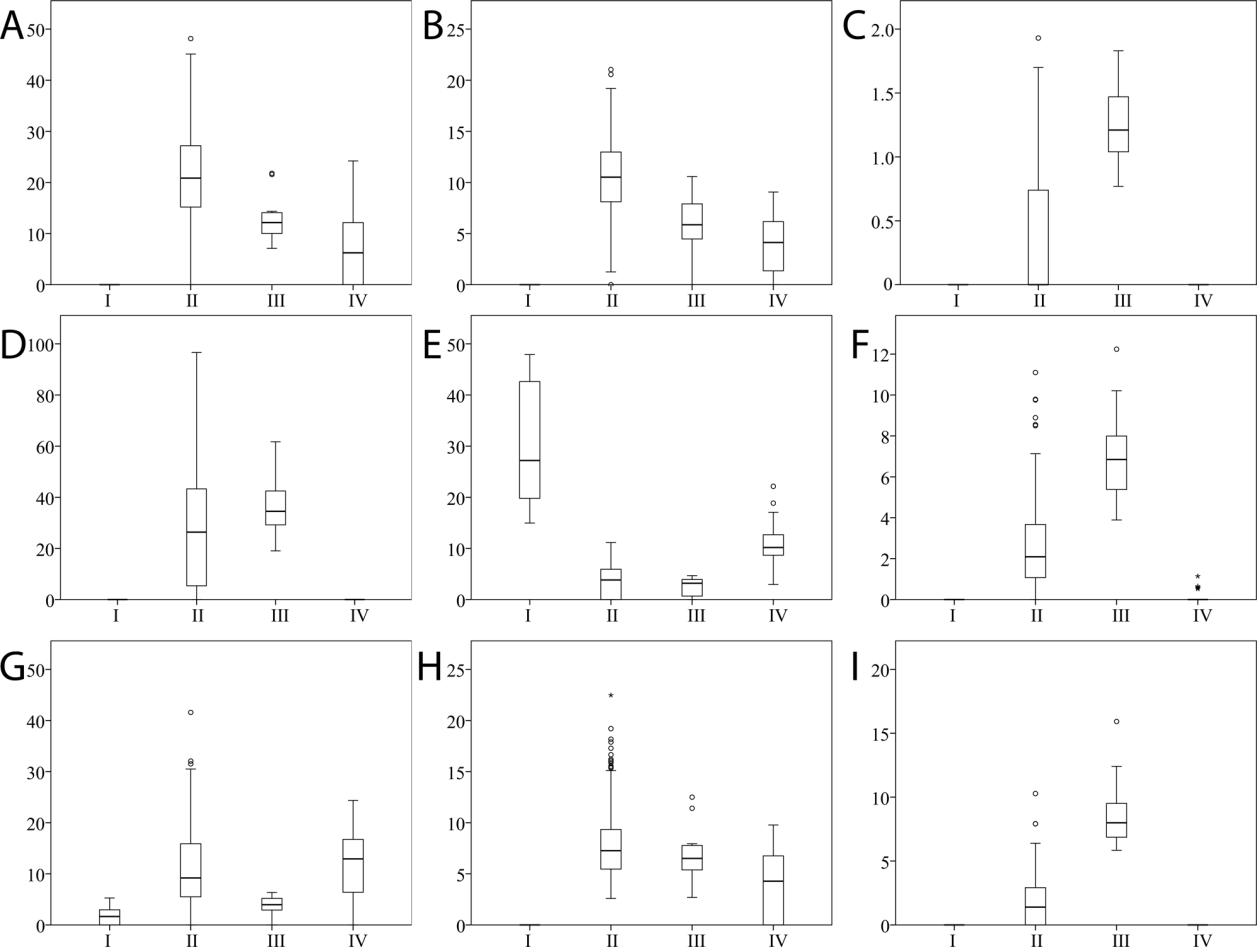
Boxplots stratified on the 4 clusters showing peak intensities across the sample population for the 9 peaks determining the clustering structure. X-axis contains the 4 clusters. Y-axis contains the peak intensity, scaled from 0 (minimum detection level) to 100 (maximum detected intensity of any peak across all samples and peaks). Peaks are listed in decreasing order of their importance for the clustering, same as in Table 2. (A) P-C peptide, Fr. 15–44. (B) P-C peptide, Fr. 1–25. (C) unidentified peak (m/z = 5980). (D) P-C peptide (1+ charge). (E) unidentified peak (m/z = 2725). (F) Cystatin B, Fr. 1–53. (G) P-C peptide, Fr. 15–35. (H) P-C peptide (2+ charge). (I) II-2 basic proline-rich protein, phosphorylated.

The Spectral Clustering co-occurrence plot (Fig 1) offers a visualization of the clustering of the participants based on their salivary peptide profiles. The clusters were designated I through IV from top-left to bottom-right of Fig 1. Cluster III contained the majority of the individuals from the study population (213, 81.6% of total), while the other three clusters were considerably smaller (cluster I– 7, 2.7% of total; cluster II– 16, 6.1% of total; cluster IV– 25, 9.6% of total). Fig 2 gives an overview of the peptide profile variation across the sample population and highlights the 9 peaks that were found by Unsupervised Feature Selection to determine the clustering (Table 2). Fig 3 shows the intensities of each of these 9 peaks in the 4 clusters. Example spectra from each cluster are shown in S3 Fig.

**Table 2:** Peaks identified by Unsupervised Feature Selection to be determining the subgroups found by Spectral Clustering. Peaks are listed in decreasing order of their effect on the clustering.

No.	Putative peak identity	Peak m/z [Da]	Peak present in [%] of individuals
**1**	P-C peptide, Fr. 15–44^a^	2921	94
**2**	P-C peptide, Fr. 1–25^a^	2523	94
**3**	unidentified peak	5980	41
**4**	P-C peptide (1+ charge)^a^	4371	69
**5**	unidentified peak	2725	77
**6**	Cystatin B, Fr. 1–53^a^	5954	79
**7**	P-C peptide, Fr. 15–35^b^	2043	92
**8**	P-C peptide (2+ charge)^a^	2185	95
**9**	II-2 basic proline-rich protein, phosphorylated^a^	7610	96

^a^Castagnola et al. 2012 [27].

^b^Identified by MALDI-TOF MS/MS.

**Fig 4:**
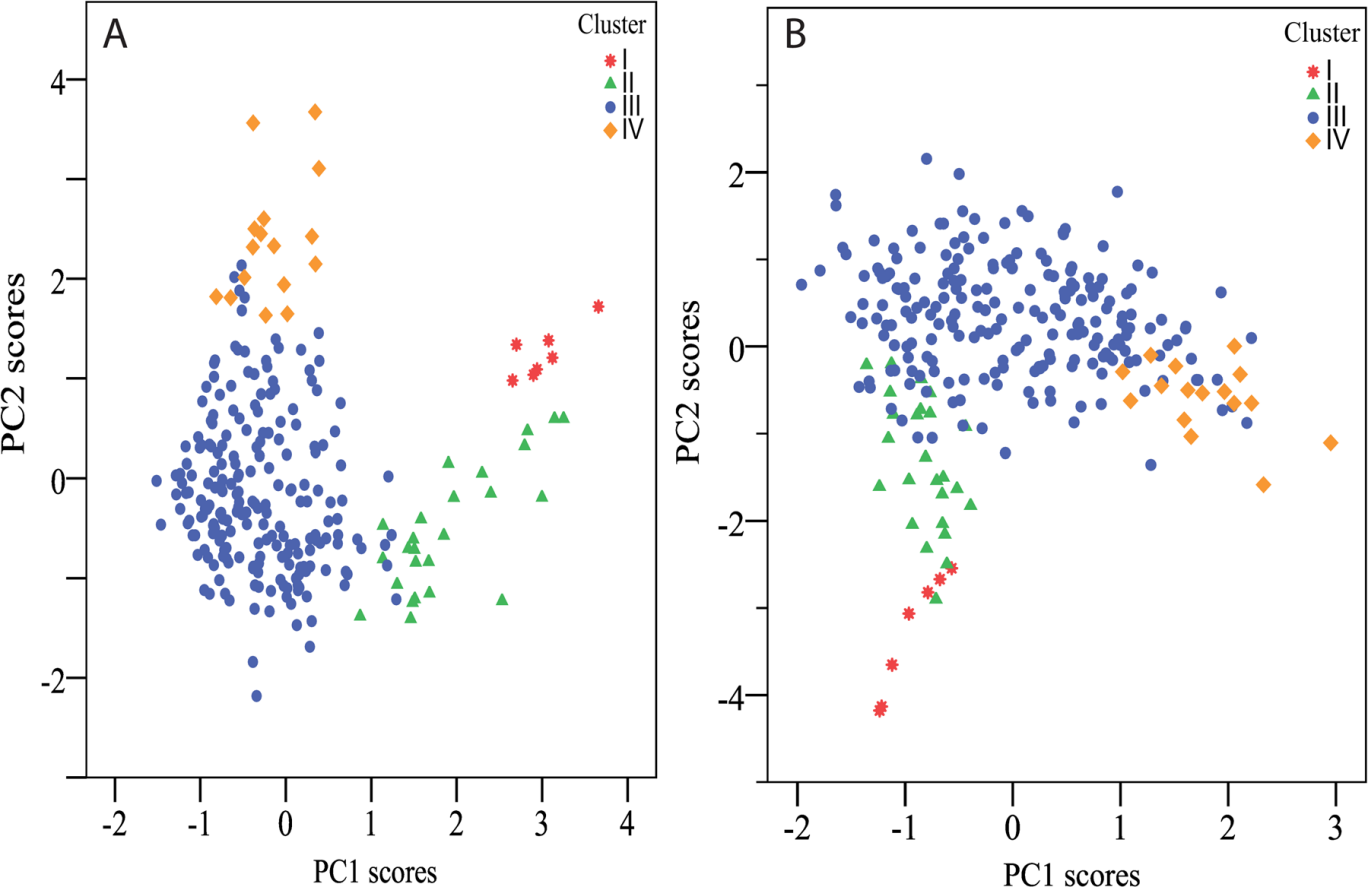
PCA scores plot. **(A) PCA based on all 80 peaks compared to (B) PCA scores plot based solely on the 9 peaks resulting from the Feature Selection procedure.** Individuals are color labeled according to their cluster. The proportion of variance explained was as follows: (A) PC1–17.4%, PC2–8.8% (26.2% cumulative). (B) PC1–39.5%, PC2–26.3% (65.8% cumulative).

Principal Component Analysis (PCA) scores plots in Fig 4 illustrate the degree to which the 9 peaks found by Unsupervised Feature Selection account for the overall grouping of individuals based on their salivary peptide profiles.

Of the 9 peaks determining the clustering of individuals based on their peptide profile (Table 2), 5 peaks were identified as either the intact form or fragments of P-C peptide. A number of significant correlations were found between these 5 peaks. First of all, there was a positive correlation (r = 0.72) between the two peaks corresponding to the 1+ and 2+ charged forms of intact P-C peptide. Secondly, there were positive correlations between the three peaks corresponding to the different P-C peptide fragments: between Fr. 1–25 and Fr. 15–44 (r = 0.75), between Fr. 1–35 and Fr. 15–44 (r = 0.51), and between Fr. 15–35 and Fr. 15–44 (r = 0.52). Finally, there was a negative correlation between the intact P-C peptide (1+ charge) and Fr. 15–35 (r = -0.63). All correlations had *p* < 0.001.

A set of nine salivary proteins with known relevance for oral health were also measured from aliquots of the same saliva samples from which the peptide profiles were acquired [17]. Salivary lysozyme and chitinase differed significantly between clusters when compared using a one-way ANOVA (both with p < 0.001) (Tables 3 and 4). No significant inter-cluster differences were found for mucins MUC5B and MUC7, salivary lactoferrin, albumin, secretory-Immunoglobulin A, cystatin S or amylase.

**Table 3:** Salivary lysozyme activity and chitinase activity stratified by Spectral Clustering clusters (Values expressed in Units of activity ml^-1^, mean ± standard error).

	I (n = 7)	II (n = 16)	III (n = 213)	IV (n = 25)	Overall (n = 261)
**Lysozyme**	400 ± 180	1,738 ± 86	2,434 ± 322	349 ± 79	1,638 ± 80
**Chitinase**	126.6 ± 55.4	28.3 ± 2.3	17.4 ± 4.2	61.0 ± 9.2	32.8 ± 2.5

**Table 4:** Differences between Spectral Clustering clusters in salivary lysozyme and chitinase activity compared using one-way ANOVA and Games-Howell post-hoc tests. The magnitude of the inter-cluster differences is quantified using Cohen’s *d* measure of effect size (*i*.*e*. the difference between the two means divided by the standard deviation of the data).

	Clusters	Cohen’s d^a^	p (post-hoc)
**Lysozyme**	I—II	1.1	0.002
	I—III	1.8	<0.001
	I—IV	0.1	0.993
	II—III	0.5	0.198
	II–IV	1.2	<0.001
	III–IV	2.4	<0.001
**Chitinase**	I—II	1.9	0.388
	I—III	1.3	0.284
	I—IV	0.7	0.793
	II—III	0.3	0.075
	II—IV	0.9	0.008
	III–IV	1.3	<0.001

## Discussion

The aim of this study was to examine the inter-individual variation in MALDI-TOF MS salivary peptide profiles and to identify potential subgroups within a population of 268 systemically healthy young adults. All volunteers were non-smokers and were screened and included in the study only in the absence of apparent caries lesions and periodontal disease. The rationale was to examine the natural variation in salivary peptide profiles of healthy individuals, a population that is commonly included as a control group in studies aiming to discover salivary biomarkers. The results of the present study suggest that individuals in a healthy population may be clustered into several sub-groups based on a limited number of MALDI-TOF MS peaks (Table 2). Furthermore, 5 of the 9 peaks found to determine the clustering were related to a single peptide. These peaks were identified as intact P-C peptide in its 1+ and 2+ charged form and 3 different fragments of P-C peptide.

P-C peptide is a member of the acidic proline-rich protein (aPRP) class of the salivary proline-rich proline (PRP) family [28]. aPRPs are secreted by the parotid as well as by the submandibular / sublingual glands and account for 20–30% of the total salivary protein content [29, 30]. aPRPs show an extensive heterogeneity and include numerous polymorphic isoforms with high sequence homology [29, 31]. aPRP precursors of P-C peptide are encoded on 2 genes: PRH1 (alleles PIF-s and Db-s) and PRH2 (alleles PRP-1 and PRP-2) [32]. Prior to secretion, these precursors are partially cleaved by proprotein convertases present in the salivary glands yielding the 44 residue-long P-C peptide (the C-terminal fragment of the precursors) and different N-terminal fragments, depending on the precursor isoform (PRP-3, PRP-4, PIF-f or Db-f) [30, 32]. The glandular convertases are unidentified metalloproteases [31, 33]. The cleavage is not complete and a proportion of the precursors are secreted intact. Interestingly, the degree of pre-secretion conversion differs between individuals but is stable for a given individual, with no day to day variation [34].

P-C peptide precursors (PRP1, PRP2, Pif-f and Db) have specific functionalities which change depending on whether they are cleaved or not, with subsequent biological consequences [33]. A 30 residue region at the N-terminal of the intact precursors is rich in aspartate, glutamine and proline and contains several serine phosphate residues. This domain allows these aPRPs to attach to recently cleaned tooth surfaces, become part of the dental pellicle and mediate hydroxyapatite crystal growth [35–38]. Moreover, the attachment causes a conformational change which exposes a bacterial binding site in the C-terminal region, such that intact precursors can also mediate the adherence of bacteria onto the tooth surface [35, 39]. Cleavage of the precursors enhances their attachment ability while removing the bacterial binding site together with the P-C peptide [33, 39]. In addition, free P-C peptide may protect against tannins, harmful polyphenolic compounds found in some plant-based foods [36]. P-C peptide effectively binds and precipitates tannins while its intact precursors do not [36].

The nomenclature of the PRP family can be confusing [40]. It has changed over time, and in some cases different names have been used in parallel for the same protein. This is particularly true for the P-C peptide, which has been designated “IB-8b” in some studies and “peptide Tz” in others, and is sometimes still classified as part of the basic PRPs rather than the aPRPs [36, 40–42]. However, all three designations refer to same peptide [43]. “P-C peptide” dominates in more recent literature and therefore this designation has been used in the present study [30, 43, 44].

The pattern of variation of P-C peptide and its different fragments in saliva may provide valuable information about the underlying state of the oral ecosystem. Un-cleaved precursors are converted post-secretion by bacterial endoproteases in the oral cavity, thus adding to the amount of P-C peptide already secreted from the glands [31]. P-C peptide itself is degraded by bacterial proteases yielding a mix of different fragments. Therefore, the relative amount of P-C peptide present in saliva and its observed pattern of fragmentation are influenced both by processes taking place within the salivary glands as well as by the particular (proteolytic) microbial profile in the oral cavity. At least 7 different P-C peptide fragments have been reported in saliva [29, 31]. The peaks of intact P-C peptide with 1+ and 2+ charge were found earlier top-down MALDI-TOF studies of salivary peptide profiles [43, 45]. A more recent study, also using top-down MALDI-TOF (with prior fractionation by nano-HPLC) found all 3 of the P-C peptide fragments selected as markers for the clusters defined in the present study (Fr. 1–25, Fr. 15–35, and Fr. 15–44) [46]. Another study, using similar techniques to examine the salivary peptide profiles of infants, found 2 of the P-C peptide fragments (Fr. 1–25 and Fr. 15–44), as well as the peaks for both the 1+ and 2+ charge intact P-C peptide [47]. All 4 peaks showed significant increases at the age of 6 months compared to the initial age of 3 months. Some intact P-C peptide is found in almost all saliva samples [29, 30]. However, the pattern of P-C peptide fragments seems to differ in healthy saliva compared to saliva from individuals with high caries experience and from patients with Sjögren’s syndrome [29]. Fr. 1–25 and Fr. 15–44 were predominant in the saliva of healthy individuals [29]. The peaks corresponding to these 2 fragments were both present in the saliva of 94% of the individuals in the present study, screened to be healthy. A previous work comparing the salivary peptide profiles of Sjögren Syndrome patients to those of healthy controls found that 2 peaks corresponding to α-defensins 1 and 2 (m/z values of 3447 Da and 3376 Da, respectively) were significantly higher in Sjögren Syndrome patients [48]. The peaks of α-defensins 1 and 2 were only detected in 8% and 5%, respectively, of the healthy adult population sampled in the current study (Table 1). P-C peptide has also been highlighted in a study examining changes in the salivary peptide profiles of children affected by type 1 diabetes compared to healthy controls [19]. The study found that intact P-C peptide was significantly higher in healthy children, while P-C peptide fragments (Fr. 1–25 and Fr. 15–44) were higher in type 1 diabetic children [19].

P-C peptide and P-C peptide fragments accounted for 5 of the 9 peaks found by Unsupervised Feature Selection to define the 4 clusters observed in the present study population (Fig 1, Table 2). Fig 4 illustrates the reliability of the Unsupervised Feature Selection results. A scores plot from a PCA analysis performed exclusively on data from the 9 selected peaks accurately maintained the grouping seen when including data from all 80 peaks.

Significant differences were found when comparing individuals belonging to the 4 different clusters with regard to salivary lysozyme and chitinase activity (Tables 3 and 4). Lysozyme is a major component of the salivary innate defense system [1, 2]. It acts by cleaving peptidoglycan, thereby killing Gram-positive bacteria *via* cell wall lysis [1, 2]. Similarly, chitinase contributes to the protection of the oral cavity from pathogenic yeast such as *Candida albicans* whose cell wall contains chitin [1, 49]. Differences in the salivary biochemistry of clusters based on salivary peptide profiles support the possibility that these profiles relate information on the underlying state of the oral ecosystem.

MALDI-TOF MS is a valuable tool for the profiling of biological samples. It is a fast, high-throughput method to obtain a molecular “fingerprint” and it can detect intact molecular species and post-translational modifications [50]. However, it also has some important limitations [51]. First of all, it is semi-quantitative. MALDI-TOF MS does not allow absolute quantification and can only quantify relative differences between samples for particular peaks. Secondly, a definitive identification of peaks by MALDI-TOF MS/MS is challenging, and for some peaks only putative identities may be obtained by matching m/z values [52]. Other top-down proteomic methods such as HPLC-ESI-MS are superior in this regard but provide lower throughput [53]. A few previous studies using top-down proteomic platforms (integrating multiple, high resolution mass-spectrometry systems and sample fractionation strategies) have compiled datasets of peptide identities, structures and m/z values that provide valuable reference points for other studies on saliva [19, 27]. These works were also used in the present MALDI-TOF MS study in the attempt to match the m/z values of unidentified peaks to known salivary peptides. While the preparation of the saliva samples in these studies (involving acidification with 0.2% TFA (1:1) prior to sample centrifugation) was different compared to the present study, this should not affect peptides in the mass range being discussed (2–12 kDa). The TFA acidification precipitates several of the large, abundant salivary proteins (e.g. amylase, mucins) while leaving the smaller proteins and peptides solubilized for analysis [43].

In summary, this study gives an overview of the peptide profile variation in the saliva of 268 healthy young adults and examines the 4 subgroups of individuals found in the study population based on these profiles. Salivary P-C peptide and its fragmentation pattern had an important role in delimiting these subgroups. Significant differences were found in the underlying functional salivary biochemistry of the subgroups with regard to the activity of lysozyme and chitinase, two enzymes relevant for oral health which are involved in the salivary innate defense system.

## Supporting Information

S1 Dataset Peaks (with m/z labels). SPSS data file containing MALDI-TOF peaks m/z and intensities for each study participant.
(SAV)

S1 Fig Exemplification of the MALDI-TOF spectra processing workflow. (A) Raw spectrum. (B) Base line subtraction and normalization for total area under the curve. (C) De-noising. (D) Peak detection.
(TIF)

S2 Fig MALDI-TOF MS/MS spectrum for P-C peptide Fr. 15–35. Peptide amino acid sequence: GPPPPPPGKPQGPPPQGGRPQ.
(TIF)

S3 Fig Example spectra from each of the 4 clusters. (A) Cluster I. (B) Cluster II. (C) Cluster III. (D) Cluster IV.
(TIF)
