# Supplementary figures and images for: A Study of the Variation in the Salivary Peptide Profiles of Young Healthy Adults Acquired Using MALDI-TOF MS
Source: PLoS One. 2016 Jun 3;11(6):e0156707. doi: 10.1371/journal.pone.0156707 (PMC4892641; doi:10.1371/journal.pone.0156707)

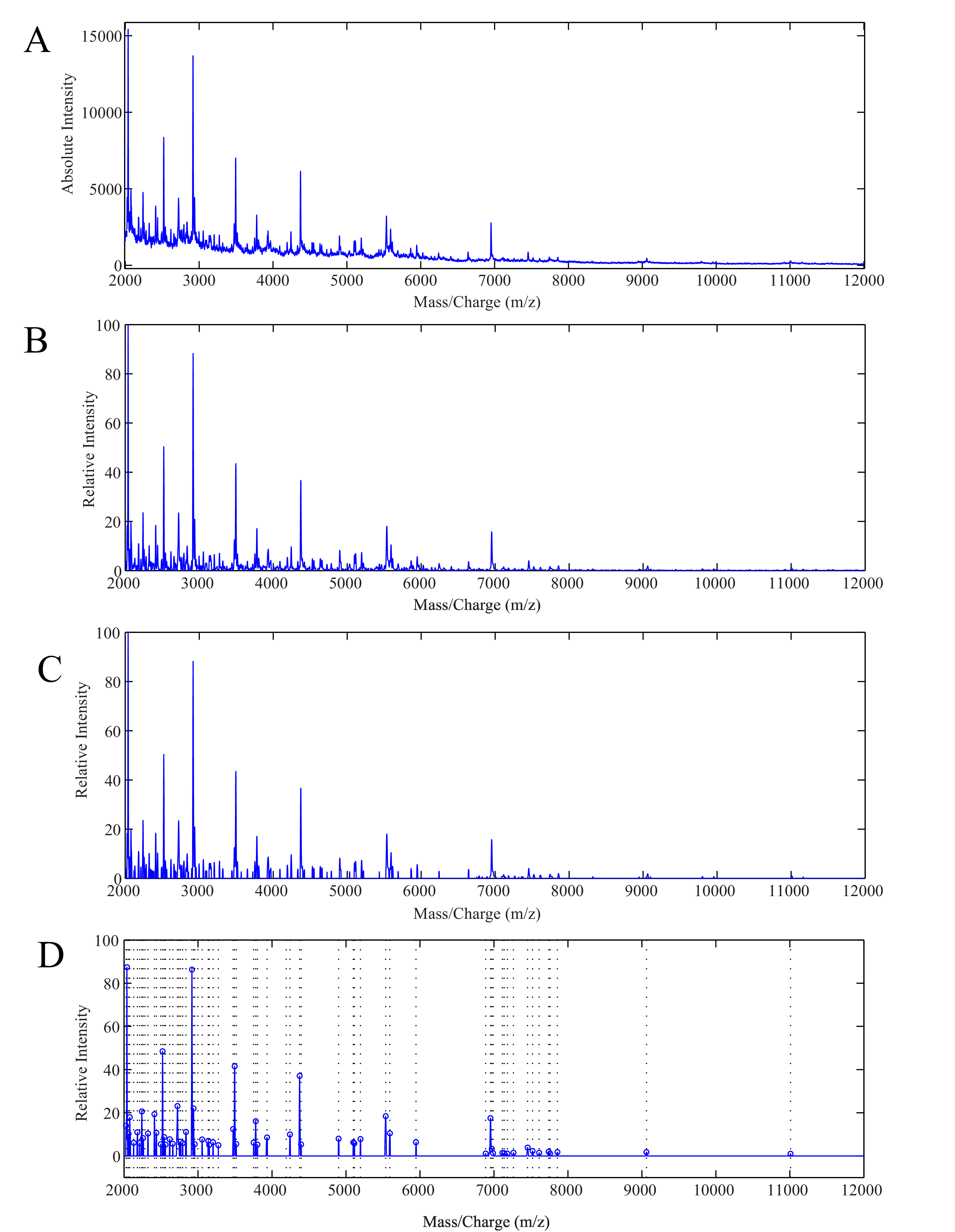

Supplement: S1 Fig — (A) Raw spectrum. (B) Base line subtraction and normalization for total area under the curve. (C) De-noising. (D) Peak detection. (TIF) [file pone.0156707.s002.tif]

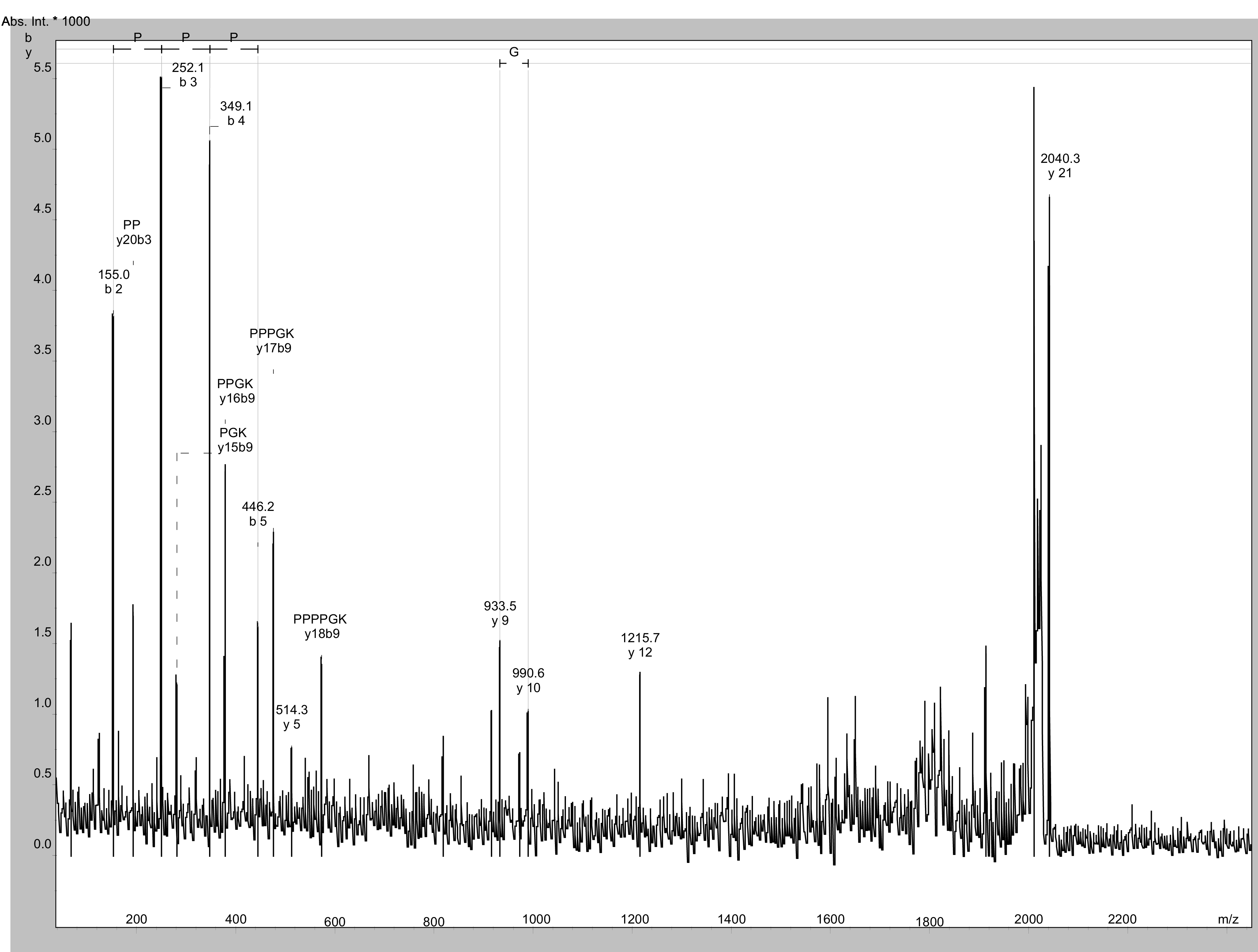

Supplement: S2 Fig — Peptide amino acid sequence: GPPPPPPGKPQGPPPQGGRPQ. (TIF) [file pone.0156707.s003.tif]

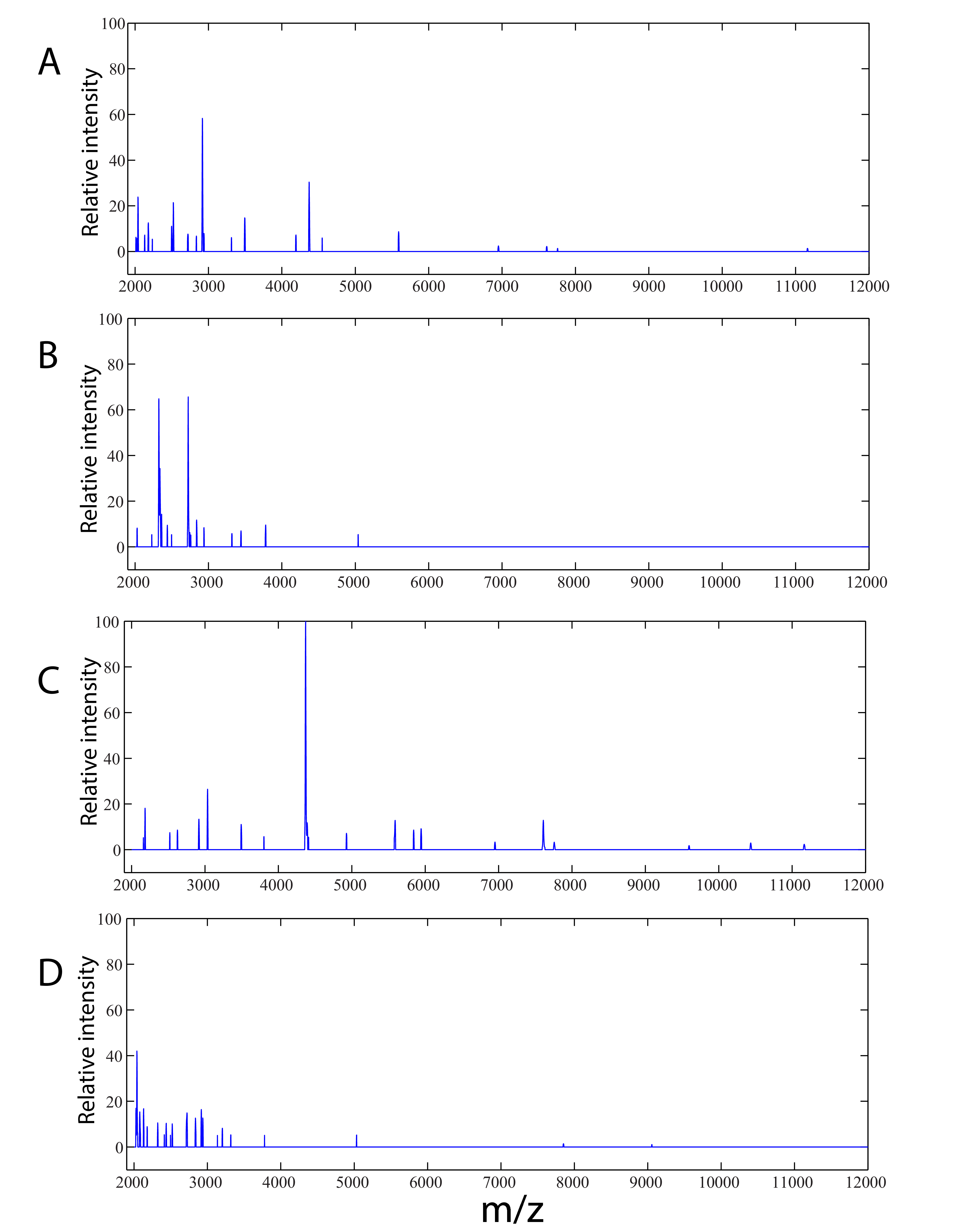

Supplement: S3 Fig — (A) Cluster I. (B) Cluster II. (C) Cluster III. (D) Cluster IV. (TIF) [file pone.0156707.s004.tif]
